# Two new species of *Paraphytis* (Hymenoptera, Aphelinidae) from Southwest China

**DOI:** 10.3897/zookeys.1021.58962

**Published:** 2021-03-01

**Authors:** Ye Chen, Hai-feng Chen, Cheng-de Li

**Affiliations:** 1 Hebei Key Laboratory of Animal Diversity, College of Life Science, Langfang Normal University, Langfang, 065000, China Langfang Normal University Langfang China; 2 School of Forestry, Northeast Forestry University, Harbin, 150040, China Northeast Forestry University Harbin China

**Keywords:** Aphelininae, Aphytini, Chalcidoidea, parasitic wasp, rainforest, taxonomy

## Abstract

Two new species of *Paraphytis* Compere, *P. bannaensis***sp. nov.** and *P. pseudovittatus***sp. nov.**, are described from the Xishuangbanna Rainforest (Southwest China). A key to species from China based on females is provided.

## Introduction

The genus *Paraphytis* was established by Compere (1925), with *Paraphytis vittatus* as the type species. Afterwards, it was treated as a synonym under *Marietta* (Compere 1936), and later as a synonym under *Aphytis* (DeBach & Rosen, 1976). It was resurrected by Kim and Heraty (2012). Currently, *Paraphytis* comprises 26 valid species (Kim and Heraty 2012; Noyes 2019). Species are distributed in the Australian, Oriental and Neotropical regions, with 11, 8 and 7 species respectively. All species of this genus with known biology are primary endoparasitoids of Diaspididae (Rosen and DeBach 1979; Kim and Heraty 2012; Noyes 2019). Rosen and DeBach (1979) provided a detailed taxonomic treatment for the species which were then placed in the *Aphytis vittatus*-group, including a key to species, descriptions or redescriptions, and photos. Kim and Heraty (2012) resurrected *Paraphytis*, and provided diagnoses to distinguish this and similar genera, including *Aphytis* and *Marietta*.

The Chinese fauna of *Paraphytis* includes five species: *P. vittatus* described by Compere (1925) from Fujian Province; *P. angustus* by Compere (1955) from Taiwan; *P. breviclavatus*, *P. densiciliatus* and *P. transversus* by Huang (1994) from Fujian Province. Herein we describe two new species and provide an identification key to Chinese species of *Paraphytis*.

## Materials and methods

In May 2019, arthropods were sampled in the rainforest canopy at the Xishuangbanna Tropical Botanical Garden in Menglun Town, Yunnan Province. Samples were obtained using a pyrethroid fog generated from a thermal fogger (Swingfog SN50, Germany, Model 2610E, Series 3). All individuals in the present study were collected from these samples.

Specimens were dissected and mounted in Canada Balsam on slides following the method described by Noyes (1982). Prior to slide mounting, specimens in ethanol were photographed with an Olympus C7070 digital camera attached to an Olympus BX51 compound microscope. Slide-mounted specimens were photographed with a digital CCD camera attached to an Olympus BX53 compound microscope. Final modifications to the images were made using Helicon Focus 6 and Adobe Photoshop CS5. Measurements were made from the slide-mounted specimens using a reticle micrometer, except for the total body length (excluding the ovipositor), which was measured from ethanol-preserved specimens before dissection. All measurements are given in micrometers (μm) except body length, which is measured in millimetres (mm). The measurements of length and width of body parts generally follows Hayat (1998), except pedicel and flagellomeres which are measured as shown in Fig. 3. Scale bars are 100 μm except where otherwise indicated. All specimens listed below are deposited in Langfang Normal University (**LFNU**), Langfang, China.

Terminology follows the Hymenoptera Anatomy Consortium (2020) for most body parts, Rosen and DeBach (1979) for bullae, and Hayat (1998) for basal cell and linea calva.

The following abbreviations are used in the text:

**F1–3** funicle segments 1–3;

**Gt_1_, Gt_2_ etc.** tergites 1, 2, etc. of gaster.

## Taxonomy

### Genus Paraphytis

Taxon classificationAnimaliaHymenopteraAphelinidae

Compere, 1925

4E3768E8-74FB-5E20-BB35-7F136C41A0F7

Paraphytis Compere, 1925: 129. Type species: Paraphytis vittata, by monotypy. Synonymy under Marietta by Compere 1936: 311; synonymy under Aphytis by DeBach and Rosen 1976: 541; revived by Kim and Heraty 2012: 544.
Syediella Shafee, 1970: 144. Type species: Syediella maculata, by original designation. Synonymy under Aphytis by Hayat 1982: 169 and under Paraphytis by Kim and Heraty 2012: 544.

#### Diagnosis.

**Figures 1–10.**
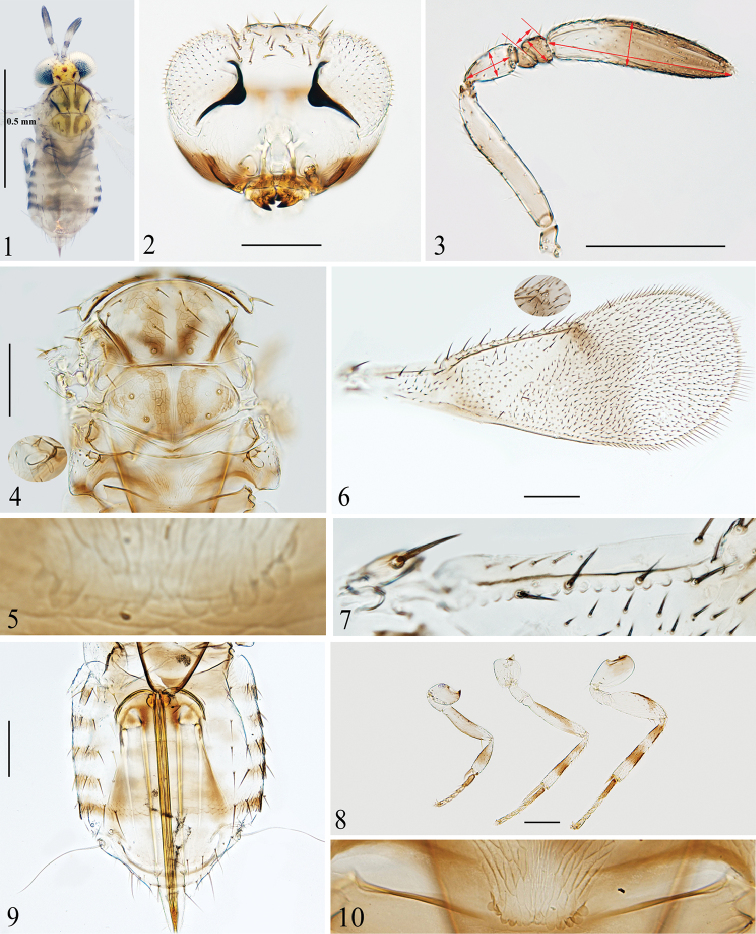
*Paraphytis bannaensis* sp. nov., holotype female (except Fig. 10) **1** body, dorsal view **2** head, frontal view **3** antenna, red arrows indicate measurements of length and width **4** mesosoma and propodeal seta **5** crenulae **6** forewing **7** submarginal vein **8** legs **9** metasoma **10** crenulae, paratype.

**Figures 11–22.**
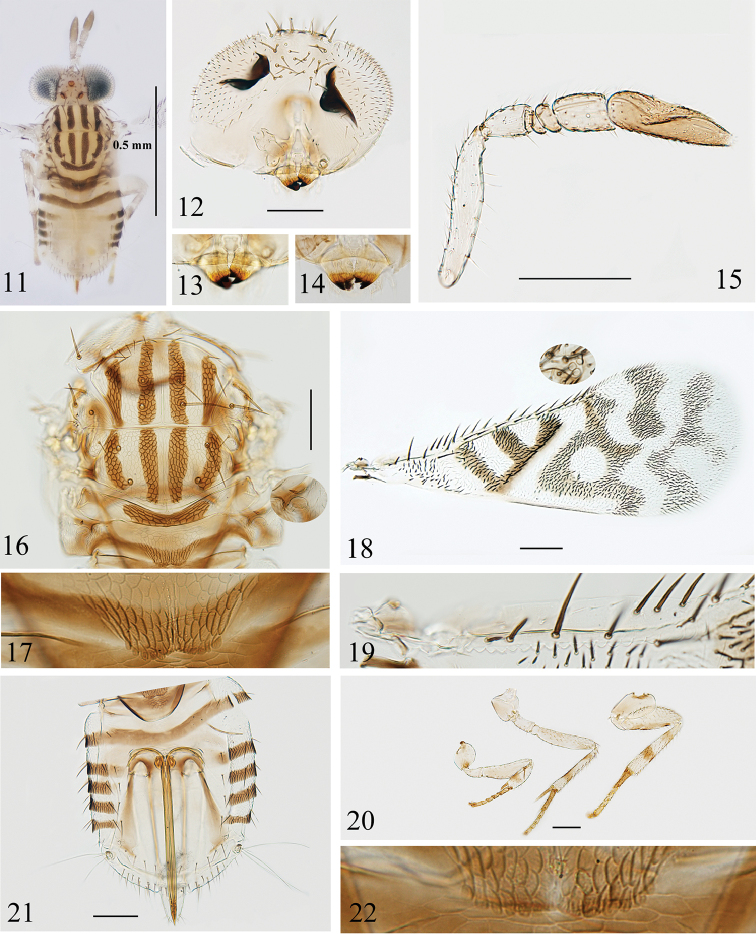
*Paraphytis pseudovittatus* sp. nov., holotype female (except Figs 14, 22) **11** body, dorsal view **12** head, frontal view **13** mandible **14** mandible, paratype **15** antenna **16** mesosoma and propodeal seta **17** crenulae **18** forewing **19** submarginal vein **20** legs **21** gaster **22** crenulae, paratype.

Species of *Paraphytis* can be recognized by the following combination of characters: antenna (Figs 3, 15) with 6 or rarely 5 antennomeres; distinctly mottled forewings (Figs 6, 18) and heavily pigmented body (Figs 1, 11); mesopleuron convex, large and undivided; axilla with one seta (Figs 4, 16); propodeum more than 2× as long as metanotum and with crenulae on posterior margin (Figs 5, 10, 17, 22); seta anterior to propodeal spiracle thin and not flattened as in *Aphytis* (Figs 4, 16; cf. fig. 243 in Kim and Heraty 2012).

##### Key to Chinese species (female) of *Paraphytis* Compere

**Table:** 

1	Antenna with 5 antennomeres	**2**
–	Antenna with 6 antennomeres	**3**
2	Clava with an incomplete transverse suture (cf. fig. 9B in Huang 1994) at about basal one third; dorsum of mesoscutum and mesoscutellum without dark longitudinal stripes; forewing mostly infuscate, with a hyaline crossband near apex (cf. fig. 9C in Huang 1994)	***P. densiciliatus* (Huang)**
–	Clava without any sutures, dorsum of mesoscutum and mesoscutellum with 2 and 4 dark longitudinal stripes respectively (Figs 1, 4); forewing with a brown band below apex of submarginal vein, and with a broad infuscated patch below stigmal vein, otherwise uniformly hyaline (Fig. 6)	***P. bannaensis* sp. nov.**
3	Body extensively pale yellow, with 4 dark longitudinal stripes (Figs 11, 16) on dorsum of mesoscutum and mesoscutellum	**4**
–	Body extensively yellow or dark; if yellow, then without any dark longitudinal stripes, at most with some dark patches (cf. fig. 11E in Huang 1994)	**5**
4	Mesoscutellum with submedian dark longitudinal stripes that do not merge with lateral stripes at posterior margin (Figs 11, 16); forewing with delta area having “F” shaped pattern formed by dark and hyaline setae and dark membrane; forewing disc with pattern posterior to linea calva formed by a transparent round patch and other irregular transparent and dark patches (Fig. 18); clava relatively slender, 3.0–3.6× as long as wide	***P. pseudovittatus* sp. nov.**
–	Mesoscutellum with submedian dark longitudinal stripes merging with lateral stripes at posterior margin (cf. fig. 265 in Rosen and DeBach 1979); forewing with delta area having an infuscated ring formed by dark and hyaline setae, without dark membrane; forewing disc with different pattern posterior to linea calva formed mainly by several subelliptical transparent patches against a dark background (cf. fig. 268 in Rosen and DeBach 1979); clava about 2.5× as long as wide	***P. vittatus* Compere**
5	Scape pale; forewing at most faintly infuscated; midlobe of mesoscutum about 1.6× as wide as long (cf. Fig. 10E in Huang 1994)	**6**
–	Scape with a dark brown oblique band apically; forewing with an “M” shaped transparent patch (cf. fig. 11C in Huang 1994); midlobe of mesoscutum 1.9× as wide as long (cf. fig. 11E in Huang 1994)	***P. transversus* (Huang)**
6	Mesofemur with a dark patch medially on outer surface; clava more than 2× as long as wide	***P. angustus* (Compere)**
–	Mesofemur pale; clava 1.8× as long as wide	***P. breviclavatus* (Huang)**

### Paraphytis bannaensis

Taxon classificationAnimaliaHymenopteraAphelinidae

Chen & Li sp. nov.

B8AE25C9-C0D4-55AD-9685-DD83C807EB72

http://zoobank.org/21D4E45E-F23E-4A0B-9824-1AF5C3B34EF8

Figs 1–10

#### Type material.

***Holotype***: ♀ [on slide, A-Pa2020011], China, Yunnan Province, Xishuangbanna, Mengla County, Menglun Town (21°54.24'N, 101°15.98'E﻿﻿, elevation ca 541 m), 13.v.2019, Zi-long Bai, Zhi-gang Chen, Cheng Wang, Hao Yu leg; deposited in LFNU. ***Paratypes***: 17♀♀ [10♀♀ on slides, A-Pa202001– A-Pa2020010; 7♀♀ in alcohol, LFNU], same data as holotype. 1♀ [on slide, A-Pa2020012, LFNU], CHINA, Yunnan Province, Xishuangbanna, Mengla County, Menglun Town (21°54.34'N, 101°16.79'E, elevation ca 618 m), 2.v.2019, Zi-long Bai, Zhi-gang Chen, Cheng Wang, Yan-feng Tong, Hao Yu leg.

#### Diagnosis.

*Paraphytis bannaensis* sp. nov. can be distinguished from other species in this genus by the following combination of characters: 5 antennomeres, midlobe of mesoscutum with two dark stripes, mostly dark brown profemur, dark tarsomeres, forewing with a brown band below apex of submarginal vein, with a broad infuscated patch below stigmal vein, and relatively long ovipositor which at least 2.0× as long as mesotibia.

#### Description.

**Female.** Holotype. Length 0.8 mm.

***Coloration*** (Fig. 1). Head mostly pale yellow, with vertex orange yellow, lower half of malar space and mouth margin dark. Occipital foramen with a dark brown transverse line on upper margin, and with lateral margins dark brown. Ocelli orange red. Setae on head dark. Antenna with funicle segments and distal half of clava dark brown, remainder yellow somewhat suffused with brown. Mandible brown with apex darker. Pronotum yellow but with central region and posterior margin dark. General coloration of mesosoma (Fig. 1) yellow, but with dark markings as followings: anterior margin of midlobe of mesoscutum and notauli; midlobe with a pair of submedian longitudinal stripes, which curve out at anterior ends; mesoscutellum with similar submedian longitudinal stripes as on midlobe, and additionally with two oblique stripes along lateral margins (not clearly visible in Fig. 1, but in the fresh specimen the lateral oblique stripes obviously merge with the submedian stripes at each posterior margin). Mesopleuron with posterior half dark brown; propodeum with lateral sides brown, and with a dark “V” shaped streak along posterior margin. Forewing (Fig. 6) with veins and posterior margin brown; disc with an oblique brown band below apex of submarginal vein, and with broad infuscated patch below stigmal vein and area around stigmal vein darker. Legs (Fig. 8) pale yellow with brown parts as followings: extreme base of procoxae; profemur dorsally except apical one third; protibiae with a broad ring basally; mesofemur with dorsal surface and distal one third; mesotibia with two rings, one near base, the other on medial area; base of metacoxae, metafemur with a curved band on dorsal surface distally, metatibia pigmented as mesotibia; all tarsomeres brown, with basitarsi darker. Metasoma (Figs 1, 9) with petiole dark, Gt_1–5_ each with pale brown to brown bands on dorsal surface medially and dark bands on lateral sides, the median bands connecting the lateral bands on Gt_1_ and Gt_5_; Gt_6_ brown, the following two tergites pale yellow. Ovipositor brown.

***Head*** (Fig. 2), in frontal view, 0.8× as high as wide; weakly reticulated. Frontovertex 0.4× head width, with two pairs of long setae, one on vertex and another along occipital margin, and with about 20 short brown setae. Ocellar triangle (Fig. 1) with apical angle 94°. Mandible bidentate. Antenna (Fig. 3) with 5 antennomeres, scape 5.0× as long as wide, slightly shorter than clava; pedicel 2.0× as long as wide, 1.2× as long as F1 and F2 combined; F1 triangular, ventral margin longer than dorsal margin, a little longer than wide; F2 trapezoidal, dorsal margin 1.6× longer than ventral margin, 0.8× as long as wide, subequal to F1 in length, 1.7× width of F1; clava slightly curved medially, 4.1× as long as wide with 7 longitudinal sensilla. Measurements, length (width): scape 112.5 (22.5); pedicel 45 (22.5); F1 17.5 (15); F2 20 (25); clava 145 (35).

***Mesosoma*** (Figs 4, 5). Dorsum of mesosoma reticulate, with sculpture on dark areas more evident. Mesoscutum with midlobe 0.6× as long as wide, about as long as mesoscutellum, and with 12 setae; lateral lobe of mesoscutum with 4 setae; axilla with 1 seta; mesoscutellum pentagonal, 0.6× as long as wide with 2 pairs of setae. Distance between anterior pair of scutellar setae 1.4× that between posterior pair. Placoid sensilla just mesad of anterior scutellar setae, and distance between sensilla about equal to distance between the posterior scutellar setae. Metanotum narrow. Propodeum with a thin seta (Fig. 4, inset) anterior to propodeal spiracle, 4.7× length of metanotum, and posterior margin with 5 (left side) + 6 (right side) crenulae (Fig. 5).

***Wings*.** Forewing (Figs 6, 7) 2.6× as long as wide, marginal setae 0.1× wing width. Costal cell 0.7× length of marginal vein, with 4 fine setae medially and 2 coarse setae apically; submarginal vein (Fig. 7) with 5 setae and 18 bullae; marginal vein with 9 setae along anterior margin; basal cell with about 17 setae in nearly 4 transverse lines. Hind wing hyaline, 5.0× as long as wide, with marginal setae 0.5× wing width. Measurements, length (width): forewing 760 (290); costal cell 150; submarginal vein 130; marginal vein 230; stigma vein 15; hind win, 600 (120); marginal setae of hind wing 60.

***Legs*** (Fig. 8). Mesotibial spur as long as corresponding basitarsus. Length measurements: mesotibia 190; mesotibial spur 75; mesobasitarsus 75.

***Metasoma*** (Fig. 9). Petiole strongly reticulated on central area just below crenulae. Gt_1–5_ with elongate reticulations on lateral sides, Gt_5_ with imbricate sculpture on dorsal surface. Setation of tergites on dorsal surface as follows: Gt_1_ with 3 setae on each side, Gt_2_ with 4 setae on left side and 3 setae on right side (4+3), Gt_3_ 5+4, Gt_4_ 4+4, Gt_5_ 4+4, Gt_6_ and Gt_7_ each with 10 setae, Gt_8_ with 4 setae. Ovipositor originating from apex of Gt_1_, 2.4× as long as mesotibia, and slightly exerted. Second valvifer 3.1× as long as third valvula; third valvula with several pale setae apically, and 1.5× as long as mesobasitarsus. Length measurements: ovipositor 450; second valvifer 340; third valvula 110.

**Male.** Unknown.

#### Variation.

Scape 5.0–6.4× as long as wide, clava 3.6–4.8× as long as wide. Forewing 2.6–2.9× as long as wide, hind wing 5.0–5.7× as long as wide. Basal cell with 15–22 setae. Posterior margin of propodeum with 5+6 to 6+7 crenulae (Figs 5, 10). Gt_2–5_ each with 7–10 setae. Ovipositor originating from apex of Gt_1_ to apex of Gt_2_, and 2.1–2.4× as long as mesotibia.

#### Remarks.

This species resembles *P. maculatus* (Shafee), with both having 5 antennomeres and similar coloration. They can be distinguished from each other by the following: midlobe of mesoscutum with only a pair of submedian longitudinal dark brown stripes, which are obviously curving out at anterior ends (vs with four longitudinal brown stripes, and with the pair of submedian stripes not curving out at anterior ends in *P. maculatus*, cf. fig. 467 in Rosen and DeBach 1979; fig. 151 in Hayat 1998; fig. 39 in Kim and Heraty 2012); legs with profemur extensively brown and all tarsomeres brown to dark brown (vs profemur pale yellow somewhat faintly suffused with dusky distally, tarsomeres mostly pale except all basitarsi and the second tarsomere of fore leg dark brown); forewing with a brown band and broad infuscated patch, without a patch of thick, darker setae in middle of proximal margin of linea calva (vs with only an oval infuscated patch below stigmal vein and with a patch of thick, darker setae in middle of proximal margin of the linea calva cf. fig. 152 in Hayat 1998); Ovipositor at least 2.0× as long as mesotibia (vs less than 2.0×).

#### Host.

Unknown.

#### Etymology.

Named after the locality of type specimen.

#### Distribution.

China (Xishuangbanna of Yunnan Province).

### Paraphytis pseudovittatus

Taxon classificationAnimaliaHymenopteraAphelinidae

Chen & Li sp. nov.

D32A37E1-1434-5E15-B73A-D7741590E0DD

http://zoobank.org/FE45DA92-F1FE-4D56-B577-3D5248ED8148

Figs 11–22

#### Type material.

***Holotype***: ♀ [on slide, A-Pa2020020], China, Yunnan Province, Xishuangbanna, Mengla County, Menglun Town (21°54.55'N, 101°16.31'E, elevation ca 570 m), 14.v.2019, Zi-long Bai, Zhi-gang Chen, Cheng Wang, Hao Yu leg; deposited in LFNU. ***Paratypes***: 1♀ [on slide, A-Pa2020021, LFNU], CHINA, Yunnan Province, Xishuangbanna, Mengla County, Menglun Town (21°53.68'N, 101°17.41'E, elevation ca 539 m), 8.v.2019, Zi-long Bai, Ye-jie Lin, Cheng Wang, Yan-feng Tong, Hao Yu leg. 1♀ [on slide, A-Pa2020022, LFNU], CHINA, Yunnan Province, Xishuangbanna, Mengla County, Menglun Town (21°54.34'N, 101°16.79'E, elevation ca 618 m), 2.v.2019, Zi-long Bai, Zhi-gang Chen, Cheng Wang, Yan-feng Tong leg. 2♀♀ [on slides, A-Pa2020023, A-Pa2020024, LFNU], CHINA, Yunnan Province, Xishuangbanna, Mengla County, Menglun Town (21°54.37'N, 101°16.71'E, elevation ca 623 m), 6.v.2019, Zi-long Bai, Ye-jie Lin, Cheng Wang, Yan-feng Tong, Hao Yu leg. 1♀ [in alcohol, LFNU], CHINA, Yunnan Province, Xishuangbanna, Mengla County, Menglun Town (21°53.59'N, 101°17.29'E, elevation ca 546 m), 4.v.2019, Zi-long Bai, Zhi-gang Chen, Cheng Wang, Yan-feng Tong, Hao Yu leg. 1♀ [on slide, A-Pa2020025, LFNU], same data as holotype.

#### Diagnosis.

*Paraphytis pseudovittatus* can be distinguished from other species in this genus by the following combination of characters: antenna with 6 antennomeres; mesoscutum and mesoscutellum each with four dark stripes; forewing with “F” shaped pattern in delta area and intricate mottled pattern posterior to linea calva (Fig. 18); F3 obviously longer than wide, clava more than 3.0× as long as wide; and posterior margin of propodeum with 6+5 to 6+6 crenulae.

#### Description.

**Female.** Holotype. Length 0.8 mm.

***Coloration*** (Fig. 11). Head mostly pale yellow, with upper margin and lateral margins of occipital foramen brown. Ocelli orange red. Setae on head dark. Antenna generally yellow, funicle segments with brownish suffusion, clava brown. Mandible (Fig. 13) with distal proximal brown to dark brown. Pronotum yellow except posterior margin dark. General coloration of mesosoma pale yellow, but with a distinctive pattern of dark markings as followings: midlobe of mesoscutum and mesoscutellum each with a pair of submedian longitudinal stripes and two stripes on lateral sides (Figs 11, 16); central region of metanotum with a transverse band; propodeum with a broad “V” shaped band along posterior margin. Forewing (Fig. 18) with delta area having “F” shaped pattern formed by dark and hyaline setae and dark membrane; disc with intricate pattern posterior to linea calva formed by a transparent round patch and other irregular transparent and dark patches (Fig. 18). Legs (Fig. 20) mostly pale yellow; coxae and femur more pale brown; tibiae with two brown rings, with basal rings at protibiae opened on dorsal surface; tarsomeres yellowish brown and with basitarsi darker. Metasoma mostly pale yellow, except the following: petiole dark except sides, with a dark band along posterior margin of Gt_1_, Gt_2–5_ with a dark patch on each side; Gt_2_ with two brownish blotches on sides interior to the dark patches; cercal plates brown; ovipositor brown.

***Head*** (Fig. 12), in frontal view 0.7× as high as wide; weakly reticulated. Frontovertex 0.3× head width, with about 30 coarse and brown setae. Ocellar triangle with apical angle 76°. Mandible bidentate (Fig. 13). Antenna (Fig. 15) with 6 antennomeres, scape 5.5× as long as wide, slightly longer than clava; pedicel 1.6× as long as wide, about as long as F3; F1 triangular, ventral margin longer than dorsal margin, about as long as wide; F2 with dorsal margin a little longer than ventral margin, 0.5× as long as wide, as long as F1; F3 cylindrical, 1.3× as long as wide, 3.4× as long as and 1.2× as wide as F2; clava 3.2× as long as wide, 2.8× the length of F3. F3 and clava each with 2 and 6 longitudinal sensilla. Measurements, length (width): scape 137.5 (25); pedicel 40 (25); F1 12.5 (15); F2 12.5 (27.5); F3 42.5 (32.5); clava 120 (37.5).

***Mesosoma*** (Figs 16, 17). Dorsum of mesosoma faintly reticulate, with sculpture on dark areas more evident. Mesoscutum with midlobe 0.6× as long as wide, about as long as mesoscutellum, and with 14 setae in 4 lines; lateral lobe of mesoscutum with 4 setae; axilla with 1 seta; mesoscutellum 0.5× as long as wide, with 2 pairs of setae. Distance between anterior pair of scutellar setae 1.3× that between posterior pair. Placoid sensilla just mesad of and slightly posterior to anterior scutellar setae, and distance between sensilla equal to distance between posterior scutellar setae. Propodeum with a thin seta (Fig. 16, inset) anterior to propodeal spiracle, 2.6× length of metanotum, and bearing 6+5 crenulae (Fig. 17) on posterior margin.

***Wings*.** Forewing (Fig. 18) 2.6× as long as wide; marginal setae 0.1× wing width. Costal cell 0.6× the length of marginal vein, with a row of fine setae and 4 coarse setae near apex; submarginal vein (Fig. 19) with 5 setae and 17 bullae; marginal vein with 10 setae along anterior margin; basal cell with about 40 dark setae. Hind wing hyaline, 4.7× as long as wide, with marginal setae 0.4× wing width. Measurements, length (width): forewing 870 (340); costal cell 170; submarginal vein 150; marginal vein 280; stigma vein 17.5; hind wing 660 (140); marginal setae of hind wing 50.

***Legs*** (Fig. 20). Mesotibial spur slightly shorter than corresponding basitarsus. Length measurements: mesotibia 210; mesotibial spur 80; mesobasitarsus 85.

***Metasoma*** (Fig. 21). Petiole strongly reticulated on central pigmented area just below the crenulae. Gt_1–5_ with reticulations on lateral sides, and bearing some setae on each reticulated area, setation as followings: Gt_1_ with 3 setae on each side, Gt_2–5_ with 4 setae on each side respectively, Gt_6_ with 10 setae between spiracles, Gt_7_ with 17 setae, Gt_8_ with 14 setae. Ovipositor originating from Gt_2_, 1.9× as long as mesotibia, and slightly exerted. Second valvifer 3.9× as long as third valvula; the latter with some hyaline setae apically, and slightly shorter than mesobasitarsus. Length measurements: ovipositor 390; second valvifer 310; third valvula 80.

**Male.** Unknown.

#### Variation.

Mandible bidentate, but a paratype specimen with mandible having a small denticulation attached to ventral tooth (Fig. 14). Scape 5.0–5.7× as long as wide, clava 3.0–3.6× as long as wide. Midlobe of mesoscutum bearing 12–15 setae. Forewing 2.4–2.7× as long as wide, hind wing 4.5–4.9× as long as wide. Posterior margin of propodeum with 6+5 to 6+6 crenulae (Figs 17, 22). Gt_8_ bearing 10–14 setae. Ovipositor 1.6–2.1× as long as mesotibia.

#### Remarks.

This species is similar to *Paraphytis vittatus* Compere in having a similar body colour. It can be separated from the latter by differences listed in the key. Apart from these differences, the new species has F3 obviously longer than wide (vs as long as or slightly wider than long), clava relatively slender, more than 3.0× (3.0–3.6×) as long as wide (vs about 2.5× as long as wide), propodeum bearing 6+5 to 6+6 crenulae on the posterior margin (vs only 4+4 to 4+5 crenulae).

#### Host.

Unknown.

#### Etymology.

From the Latin prefix *pseudo*-, and *vittatus* reference to the fact that this species is easily confused with *P. vittatus*.

#### Distribution.

China (Xishuangbanna of Yunnan Province).

## Supplementary Material

XML Treatment for Paraphytis

XML Treatment for Paraphytis bannaensis

XML Treatment for Paraphytis pseudovittatus
